# Detrimental effect of cardiopulmonary bypass(CPB) on malignant disease

**DOI:** 10.1186/1749-8090-6-13

**Published:** 2011-02-04

**Authors:** Ahmad K Darwazah, Saleh Shehata

**Affiliations:** 1 Department of Cardiac Surgery, Makassed Hospital, Jerusalem, Israel

## Abstract

Patients with coronary artery disease associated with malignancy are a difficult group of patients to treat. The ideal approach to manage them is still controversial. Both problems can be manage by either a combined or staged operation. The use of CPB during revascularization of the myocardium among patients with malignant disease, may have an effect on dissimination of malignant cells. This was observed among two of our patients. We believe that the use of off-pump technique to revascularize the myocardium is a safe approach and can be performed either in combined or staged surgery to resect malignant disease.

## Commentary

Patients with coronary artery disease associated with malignancy are a difficult group of patients to treat. The ideal approach to manage them is still controversial. Both problems can be managed by either a combined or staged operation. The real issue lies in the technique of bypass used to revascularize the myocardium. We and others proved that off-pump technique is a safe approach used to revascularize the myocardium which can be done with excision of the tumour at the same time or in a staged surgery [1,2].

The effect of CPB on malignant cell growth and dissimination is not known. It is well documented that the use of CPB has a direct inhibitory effect on both cell mediated and humoral immunity [3] which subsequently may affect the spread of malignant cells.

The use of standared CPB to revascularize the myocardium among these patients showed contradicting results. Most of the studies found that this technique is safe and efficient when used in combined surgery to treat both lung cancer and myocardial revascularization [4,5]. Others,found that some of their patients had widespread malignancy after successful operation [6].

Recently, we were confronted with two cases with coronary artery disease who needed surgical myocardial revascularization. One patient had a localized moderately differentiated adenocarcinoma of the sigmoid colon and the other patient had no known malignancy. Both patients underwent successful myocardial revascularization using conventional CPB. During follow up, the patient with colonic cancer who was scheduled to undergo surgery came back after 3 weeks with widespread malignancy affecting the abdominal, mediastinal, and axillary lymph nodes together with bilateral pulmonary metastasis. The other patient was seen with recurrent right sided pleural effusion one month after surgery. Initially, it was thought to be a sequale of cardiac operation. Aspiration and cytology showed evidence of metastatic malignant cells with unknown primary.

Our observation emphasized that CPB may have a direct effect on the spread of malignant cells and even it may stimulate the growth of hidden malignancy. The exact cause why malignant cells in our cases behaved in that manner?. A previous study by Tonnesen and coworkers [7] showed that the natural killer cells (N K cells)which have spontaneous cytotoxicity against tumour cells are inhibited during and after cardiopulmonary bypass. Also the complement factors, lymphocytes, and neutrophils are depleted. All these factors may explain the spread of malignant cells.

We believe that CPB should be avoided among patients with combined coronary artery disease and malignancy unless both diseases are treated at the same time. The use of off-pump bypass to revascularize the myocardium together with excision of the tumour in either a combined or staged approach is a safe technique to avoid the spread of malignant cells.

## References

[B1] Darwazah AK Osman M Sharabati B Use of off-pump coronary artery bypass surgery among patients with malignant disease J Card Surg 2009 in press 19210552 10.1111/j.1540-8191.2008.00778.x

[B2] Dyszkiewicz W Jemielity MM Piwkowski CT Simultaneous lung resection for cancer and myocardial revascularization without cardiopulmonary bypass (off-pump coronary artery bypass grafting) Ann Thorac Surg 2004 77 1023 1027 10.1016/j.athoracsur.2003.07.041 14992919

[B3] Knudsen F Andersen LW Immunological aspects of cardiopulmonary bypass J Cardiothorac Anesth 1990 4 245 258 10.1016/0888-6296(90)90246-C 2131874

[B4] Rao V Todd TRJ Weisel RD Results of combined pulmonary resection and cardiac operation Ann Thorac Surg 1996 62 342 346 10.1016/0003-4975(96)00206-8 8694588

[B5] Miller DL Orszulak TA Pairolero PC Combined operation for cancer and cardiac disease Ann Thorac Surg 1994 58 989 993 10.1016/0003-4975(94)90442-1 7944820

[B6] Tanaka H Narisawa T Hirano J Efficacy of off-pump coronary artery bypass grafting in patients requiring noncardiac operation Kyobu Geka 2001 54 1107 1111 11761894

[B7] Tonnesen E Brinklov MM Christensen WJ Natural killer cell activity and lymphocyte function during and after coronary artery bypass grafting in relation to the endocrine stress response Anesthesiology 1987 67 526 533 10.1097/00000542-198710000-00014 3499099

